# Preparation and Performance of Sintered Fe-2Cu-2Mo-0.8C Materials Containing Different Forms of Molybdenum Powder

**DOI:** 10.3390/ma12030417

**Published:** 2019-01-30

**Authors:** Wenchao Chen, Jigui Cheng, Pengqi Chen, Jianhua Zhang, Bangzheng Wei

**Affiliations:** 1 School of Materials Science and Engineering, Hefei University of Technology, Hefei 230009, China; wcchen88@126.com (W.C.); jhzhang151@126.com (J.Z.); bzwei90@163.com (B.W.); 2 Research Centre for Powder Metallurgy Engineering and Technology of Anhui Province, Hefei 230009, China

**Keywords:** ferrous powder metallurgy, Mo-containing powder, mechanically alloying, warm compaction, wear-resistance property

## Abstract

Fe-2Cu-2Mo-0.8C powder mixtures were prepared by mixing Fe, Cu and C elemental powders with different forms of Mo-containing powder (pure Mo powder, prealloyed Mo-Fe powder and mechanically alloyed Mo-Fe powder, respectively). The powder mixtures were warm pressed under different pressures and temperatures. Properties of the green compacts and the sintered parts were tested to investigate the effects of the different ways of introducing molybdenum. The test results show that a green density of 7.32 g/cm^3^ was obtained for Fe-2Cu-2Mo-0.8C powder mixtures containing mechanically alloyed Mo-Fe powders, under a warm compaction pressure of 800 MPa and warm pressing temperature of 120 °C, respectively. The sintered Fe-2Cu-2Mo-0.8C specimens added with mechanically alloyed Mo-Fe powders had a density of 7.31 g/cm^3^, a hardness of 95 HRB and a tensile strength of 618 MPa, respectively. Compared with the sintered samples, added Mo in the forms of pure Mo and prealloyed Mo-Fe powder, the sintered parts added with mechanically alloyed Mo-Fe powders had more uniform microstructure, better mechanical and wear-resistant properties.

## 1. Introduction

Ferrous powder metallurgy (PM) parts are now widely used in automotive, machinery, appliances, military and many other applications [1]. With the developments of advanced manufacturing, there is an emerging need for ferrous powder metallurgy parts with high performance [2,3]. Iron-copper-carbon (Fe-Cu-C) alloy is the most widely used ferrous material, which possesses more than 50% in the sintered ferrous PM parts. Ferrous parts containing about 2–5 wt.% of copper and 0.6–1 wt.% of carbon are commonly used in automotive applications (e.g., valve seat rings and guides, shaft sleeves, lifter parts, pump gears, etc.) [4]. 

To further improve the properties of Fe-Cu-C PM parts, Mo has been extensively investigated as an alloying element because of its excellent solution strengthening ability, which can also refine the grain size of the pearlite compared with other alloying elements, such as Mn, Ni, and Cr [5,6]. Yoo et al. studied the sintered properties of Fe-Cu-C PM steel with high carbon and elemental Mo addition and the samples own moderate mechanical properties, finding good wear resistance due to high sintering densification and solid solution strengthening [7]. Rathore et al. prepared ferrous alloys containing Mo and Cu, and found that adding of elemental Mo and Cu can increase the tensile strength and hardness of the sintered samples, but the ductility and impact strength of the specimens decreased [8].

Although it has been shown that the addition of some alloying elements with high hardness can increase the mechanical properties of ferrous PM parts, when added in the form of pure metal powder, the high hardness of these metal powders may lower the compressibility of the powder mixtures, resulting in the decrease of density of the compacts and the sintered parts [9,10,11]. Therefore, in recent years, researchers have tried introducing alloying elements into the ferrous parts in the form of prealloyed powder and some other forms. These methods can improve the homogeneity of the powder mixtures, reduce oxidation of the raw powder and facilitate diffusion homogenization during sintering [12,13]. 

It is also desirable to reduce the residual pores in a sintered ferrous structure part to increase its mechanical properties. By now, many methods have been tried to prepare ferrous PM parts with high density (low porosity), such as the repressing-resintering method, the warm compaction method, the infiltration method, the sinter-forging method and so on [14,15,16,17]. Among these methods, warm compaction has been shown to be one of the most attractive processes for preparing ferrous structural parts with a high density and an excellent property at relatively low-cost. During warm compaction, the powder mixtures, containing some special lubricants, are heated to a pre-set temperature and pressed in a warm die. After pressing, compacts with a high density can be obtained [18,19]. 

In this paper, three different kinds of Mo-containing powders were blended with iron, copper, graphite powder and composite lubricant to obtain Fe-2Cu-2Mo-0.8C powder mixtures for warm compaction. The powder mixtures were subsequently warm compacted at different pressures and the green compacts were sintered to obtain ferrous parts. The compactability of the powder mixtures containing different forms of the Mo element, density, microstructure and mechanical properties of the sintered samples were systematically tested to investigate the effects of Mo addition forms on the properties of the powder mixtures and performances of the sintered samples. 

## 2. Materials and Methods

The reduced iron powder (average particle size of 75 μm), electrolytic copper powder (average particle size of 32 μm) and flaky graphite powder (average particle size of 28 μm) were used as raw materials. The molybdenum element was added in the forms of pure Mo powder (average particle size of 12 μm), prealloyed Mo-Fe powder (average particle size of 17 μm) and mechanically alloyed Mo-Fe powder (average particle size of 18 μm), respectively. The molybdenum content in the prealloyed Mo-Fe powder and the mechanically alloyed Mo-Fe powder was about 70 wt.%. The mechanically alloyed Mo-Fe powder was prepared by high-energy ball milling 70 wt.% Mo powder and 30 wt.% Fe powder. The commercially available Fe powder was ball milled with the Mo powder on a planetary ball mill (QM-3SP24, Nanjing Chishun Science & Technology Co., Ltd., China) at a speed of 400 rpm for 4 h with a ball-to-powder weight ratio of 3:1.

The above powders (Fe, Cu, C and Mo-containing powder) were mixed in a V-type mixer for 2 h. The composition of the obtained powder mixtures were 96.2 wt.% Fe, 2.0 wt.% Cu, 2.0 wt.% Mo and 0.8 wt.% C (Fe-2Cu-2Mo-0.8C). A homemade composite lubricant composed of stearate, amide waxes and some other gradients were added into the powder mixtures during blending with a weight amount of 0.6 wt.%. During warm compaction, the powder mixtures were pressed in a heated steel die at about 120 °C and pressure of 500 to 800 MPa uniaxially. The green compacts were subsequently sintered at 1150 °C in a H_2_ atmosphere for 90 min without pressure. The theoretical density of the Fe-2Cu-2Mo-0.8C samples were estimated according to the addition law between the quality and composition. 

X-ray diffraction analysis (XRD, D/MAX2500V, Rigaku, Tokyo, Japan) was performed on the Mo-containing powders to identify the phase composition with a scanning range of 10–90°. The density of green compacts and sintered samples was measured by the Archimedes’ method according to ASTM B328-2003 standards. 

The microstructure and chemical composition characterization were carried out using a scanning electron microscope (SEM, JSM-6490LV, JOEL, Tokyo, Japan) and energy-dispersive spectroscopy (EDS). The content of carbon was tested by a carbon and sulfur analyzer (CS-3000, NCS Testing Technology Co. Ltd., Beijing, China). The tensile strength test was performed using a universal material testing machine (MTS-809, MTS Systems Corporation, Eden Prairie, MN, USA) by the dumbbell specimen with a length of 90 mm at a crosshead speed of 0.5 mm/min. The hardness was measured using the Rockwell Hardness Tester (HR-150A, Shanghai Yanrun Co. Ltd., Shanghai, China) and was performed under a load of 100 kg at room temperature. For each test, three samples were tested to ensure repeatability. The wear tests were carried out using an end face friction and wear tester. Cr12 with a hardness of 58–60 HRC were used as the counter material. The wear tests were conducted at a fixed load of 100 N, with the plate rotated at 1 m/s for 5 min.

## 3. Results and Discussion

### 3.1. Characterization of the Powders

**Figure 1:**
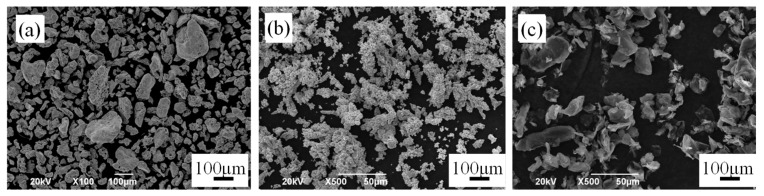
SEM images of the iron powder (**a**), copper powder (**b**) and graphite powder (**c**).

Figure 1 shows SEM images of the different raw powders. It can be seen that particles of the reduced iron powder (Figure 1a) had a wide particle size distribution and coarse surface with many micropores. The electrolytic copper powder (Figure 1b) and graphite powder (Figure 1c) had irregular and flaky shapes, respectively. 

**Figure 2:**
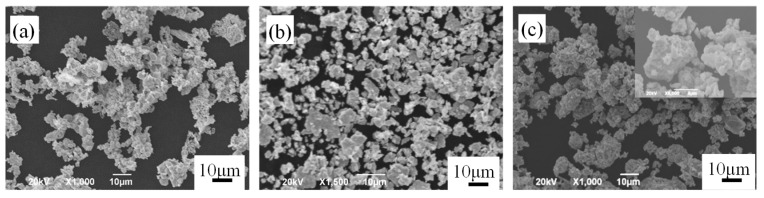
SEM images of the pure Mo powder (**a**), prealloyed Mo-Fe powder (**b**) and mechanically alloyed Mo-Fe powder, the inset is the magnification (**c**).

Figure 2 shows the morphologies of different Mo-containing powders added in the powder mixtures. The morphologies of the pure Mo (Figure 2a), prealloyed Mo-Fe powders (Figure 2b) and mechanically alloyed Mo-Fe powder (Figure 2c) were of a granular shape. For the mechanically alloyed Mo-Fe powder, small molybdenum particles attached to the iron particles.

**Figure 3:**
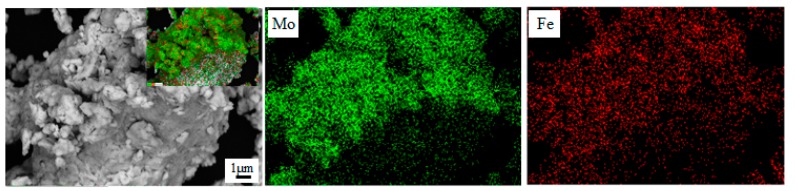
SEM images with energy-dispersive spectroscopy (EDS) analysis of the mechanically alloyed Mo-Fe powder.

Figure 3 shows the SEM images with the EDS analysis of the mechanically alloyed Mo-Fe powder. As shown in Figure 3, the EDS analysis confirmed that the Mo powders were attached to Fe particles, on the surface of the iron particles, and that the Mo particles had a homogeneous distribution. 

**Figure 4:**
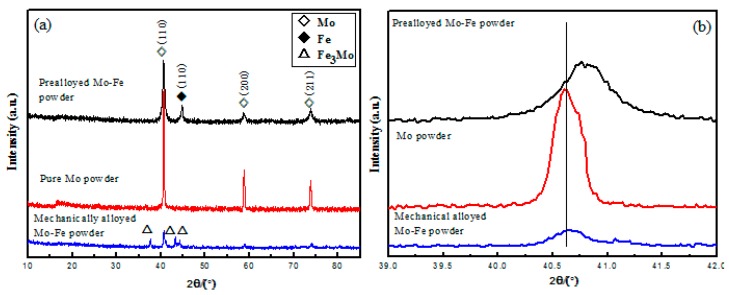
XRD patterns of the three different Mo-containing powders (**a**) and the magnification image for (110) crystal face (**b**).

Figure 4 shows XRD patterns of the different Mo-containing powders. The patterns could be indexed to well-crystalline Mo (JCPDS No. 42-1120, marked by white squares) and Fe (JCPDS No. 89-7194, marked by black squares). It is also shown that the diffraction peaks (110) of molybdenum for the prealloyed Mo-Fe powder and mechanically alloyed Mo-Fe powder shifted to a large angle, which may have been caused by the diffusion of iron to molybdenum [20,21]. The prealloyed process caused a greater diffusion of Fe, and the mechanically alloyed process caused a partial diffusion of iron, which could have made diffusion bonding of small molybdenum powder to iron. During mechanical alloying, same ferromolybdenum compounds were generated and diffraction peaks of the Fe_3_Mo phase, marked by white triangles, are found in the XRD patterns.

### 3.2. Effects of Mo Addition Forms on the Properties of Green Compacts 

**Figure 5:**
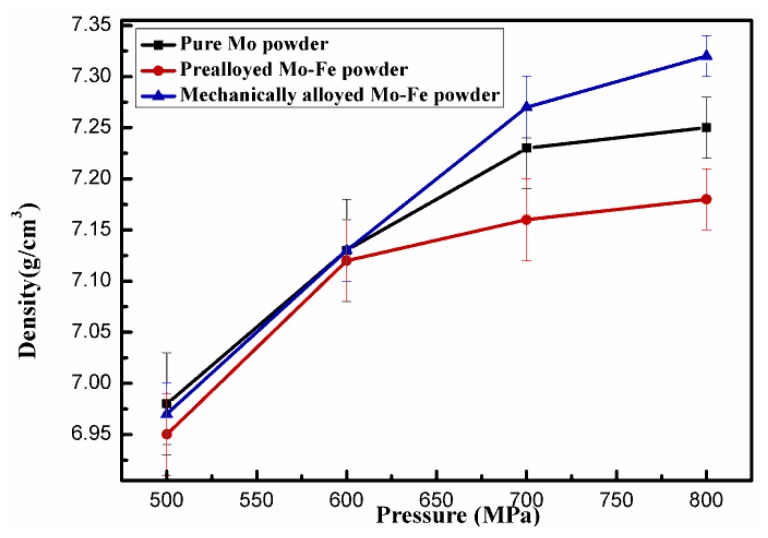
Green density of the Fe-2Cu-2Mo-0.8C compacts from powder mixtures containing different forms of Mo as a function of compaction pressure of 500–800 MPa.

Figure 5 shows the green density of Fe-2Cu-2Mo-0.8C compacts by warm pressing the powder mixtures added with different forms of Mo at a pressure of 500–800 MPa and a temperature of 120 °C. The theoretical density of the Fe-2Cu-2Mo-0.8C samples was 7.78 g/cm^3^. It was obvious that the powder mixtures containing mechanically alloyed Mo-Fe powder had the highest compressibility, and a maximum green density of 7.32 g/cm^3^ was obtained at a compaction pressure of 800 MPa. The green density from powder mixtures added with pure Mo powder and the prealloyed Mo-Fe powder was 7.25 g/cm^3^ and 7.18 g/cm^3^ at the same pressure, respectively. Figure 5 shows that the addition form of the Mo powder also had a great influence on the compressibility of the Fe-2Cu-2Mo-0.8C powder mixtures. When molybdenum was introduced in the forms of pure Mo and prealloyed Mo-Fe powder, the powder mixtures had low plasticity and were easy to aggregate, which resulted in the low green density of the powder compacts. However, when molybdenum was introduced in the forms of mechanically alloyed Mo-Fe, the powder kept the compressibility of the reduced iron powder and fine molybdenum powder, which stuck to the surfaces of iron powder. So the powder mixtures containing mechanically alloyed Mo-Fe powder had flowability and higher compressibility.

### 3.3. Microstructure and Property of the Sintered Fe-2Cu-2Mo-0.8C Specimens

**Figure 6:**
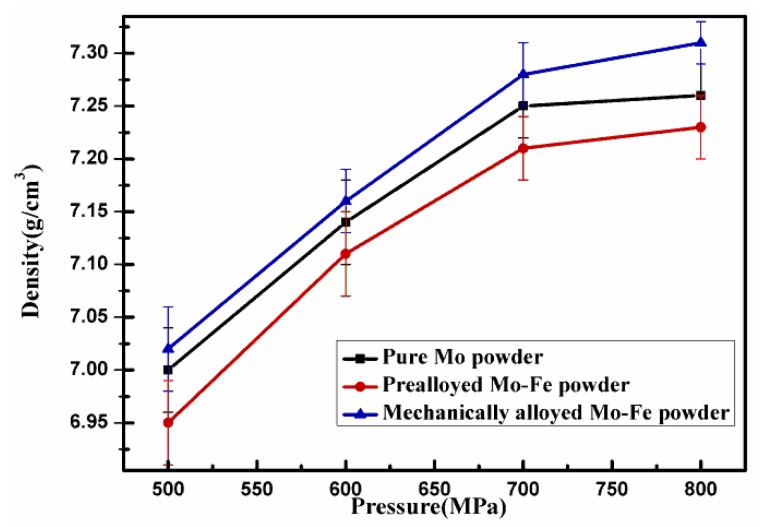
Density of the sintered Fe-2Cu-2Mo-0.8C samples from powder compacts containing different forms of Mo.

Figure 6 shows the density of the Fe-2Cu-2Mo-0.8C samples sintered at 1150 °C in a H_2_ atmosphere for 90 min. From Figure 5 and Figure 6, it can be seen that the compaction pressure also influenced the sintered density of the samples. Furthermore, Figure 5 shows that the sintered specimens prepared from powder mixtures containing the mechanically alloyed Mo-Fe powder had a maximum density of 7.31 g/cm^3^, but this value for powder mixtures containing the pure Mo and prealloyed Mo-Fe powder was only 7.23 g/cm^3^ and 7.26 g/cm^3^, respectively. Again, the advantages of introducing the Mo element in the form of a mechanically alloyed state have been shown.

The measured results for sintered density of the different Fe-2Cu-2Mo-0.8C samples revealed that introducing forms of Mo, due to the difference of the Mo form existing in raw powders, had an influence on the sintering densification process. This influence may have been caused in following ways: 

(1) Due to the high density of the green compacts, the Fe-2Cu-2Mo-0.8C samples containing the mechanically alloyed Mo-Fe powder showed the maximum sintered density value. During sintering, the particles started to grow and fill the pores inside the dense substance, so the green compacts with low porosity were conducive to obtain sintering samples with a higher density [22].

(2) In the sintering process, molybdenum was transferred from the high concentration part (particle surface) to the low concentration part by solid phase diffusion, and pores were formed in the original position of molybdenum particles [23]. For the mechanically alloyed Mo-Fe powder, the Mo particles were small and dispersed, and the pores generated by diffusion during sintering densification were more likely to disappear. At the same time, in this experiment, partial diffusion of molybdenum formed in the process of high-energy ball milling reduced the solid phase diffusion distance and promoted sintering densification. Combined with the high density, this was beneficial to improve the mechanical properties of the sintered samples.

**Figure 7:**
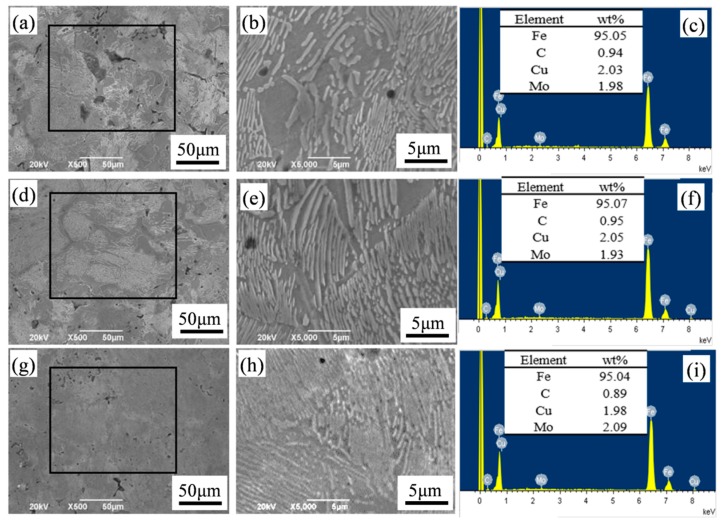
SEM images and EDS results of the sintered Fe-2Cu-2Mo-0.8C samples added with different forms of Mo: (**a**–**c**) Pure Mo powder; (**d**–**f**) prealloyed Mo-Fe powder; (**g**–**i**) mechanically alloyed Mo-Fe powder.

Figure 7 shows the surface SEM image and EDS results of the sintered Fe-2Cu-2Mo-0.8C sample. Compared with pure Mo (Figure 7a,b) and prealloyed Mo-Fe powder (Figure 7d,e), the microstructure of sintered sample with mechanically alloyed Mo-Fe powder (Figure 7g,h) was more uniform, and the pearlites were smaller and extended into the ferrite grain significantly. This was because, under the action of high-energy ball grinding, molybdenum powder adheres to the surface of iron particles and spreads evenly in the sintered body structure. And the mechanical alloying process caused partial diffusion of iron and molybdenum, which was conducive to the diffusion of the Mo element in the sintering process. This obvious change promoted the grain refinement and made the grain size of ferrite become smaller. According to the EDS results, the final chemical composition of the sintered samples consisted of about 2 wt.% Fe, 2 wt.% Cu, and 2 wt.% Mo, and the carbon content of the sintered samples prepared from pure Mo, prealloyed Mo-Fe powder and mechanically alloyed Mo-Fe powder were 0.782, 0.771, 0.779 wt.%, respectively. This result showed that there was no obvious change in Fe-2Cu-2Mo-0.8C composition.

**Figure 8:**
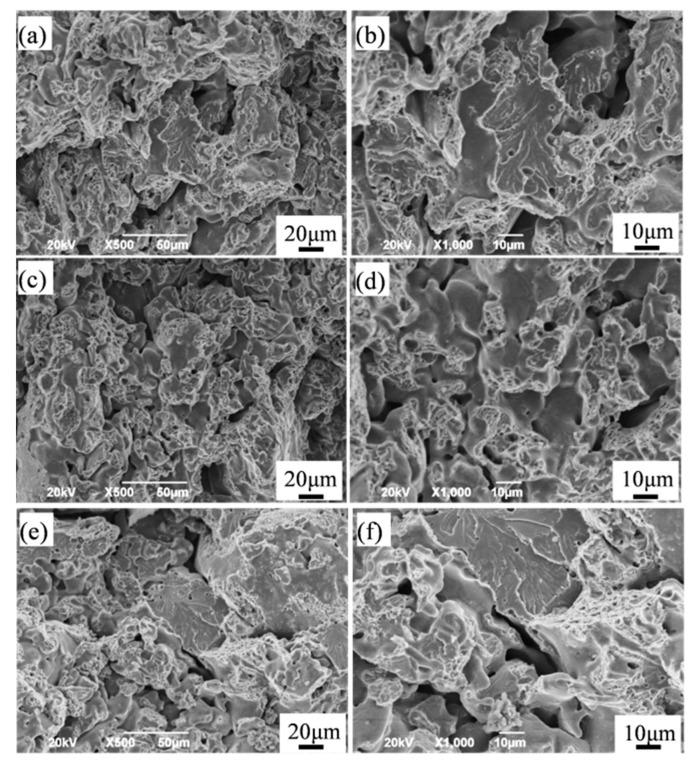
Fracture surfaces of the sintered Fe-2Cu-2Mo-0.8C samples added with different forms of Mo: (**a**) Pure Mo powder; (**b**) prealloyed Mo-Fe powder; (**c**) mechanically alloyed Mo-Fe powder.

Figure 8 shows the fracture surfaces of the sintered Fe-2Cu-2Mo-0.8C samples added with different forms of Mo. After fracturing, the closed pores could be observed on the fracture surface. The fracture surface of samples containing pure Mo (Figure 8a,b) and prealloyed Mo-Fe powder (Figure 8c,d) consisted of a small number of dimples and plenty of pores (including obturation, due to fracture). The microcrack initiation tended to occur in the areas which consisted of pores, which propagated quickly. The fracture surface of the sintered samples with mechanically alloyed Mo-Fe powder (Figure 8e,f) showed the characteristics of the mixed fracture—that in addition to the dimples—intercrystalline and transcrystalline cleavage fractures were also observed, which resulted in the increased strength. Moreover, this fracture had fewer pores and a more uniform tissue.

Table 1 lists the tensile strength and hardness of the sintered Fe-2Cu-2Mo-0.8C specimens added with different forms of Mo. A maximum tensile strength of 618 MPa and hardness of 95 HRB were obtained for the specimens containing mechanically alloyed Mo-Fe powder. When Mo was added in the form of mechanically alloyed Mo-Fe powder, molybdenum and iron powder in the fine powder mixtures contacted well and partially diffused during high-energy ball milling. This was beneficial to the solid-state diffusion during sintering, and promoted the densification and the formation of pearlite, which increased the mechanical properties of the sintered samples. 

**Figure 9:**
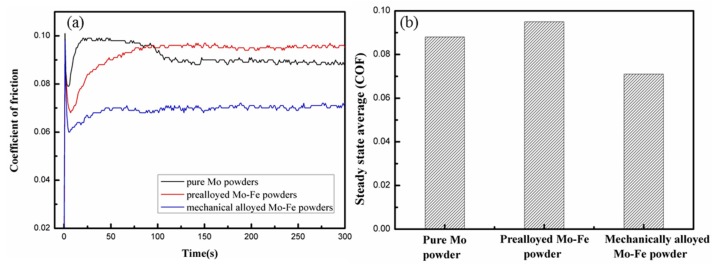
Coefficient of friction (**a**) and steady state COF (**b**) of the samples containing different forms of Mo powder.

The influence of the Mo addition forms on coefficient of friction (COF) is shown in Figure 9a and the corresponding steady-state coefficient of friction is shown in Figure 9b. Due to the different microstructural features, and the hardness and strength values, the samples added with varied Mo addition forms showed a different friction behavior in the wear test. The samples containing prealloyed Mo-Fe powder had the highest COF value (0.095) and the wear rate was 9.72 × 10^−4^ mm^3^∙N^−1^∙m^−1^ while the samples with mechanical alloyed Mo-Fe powder showed the lowest COF (0.071) and the wear rate was 7.52 × 10^−4^ mm^3^∙N^−1^∙m^−1^, which took the shortest time to reach the wear stability stage. 

**Figure 10:**
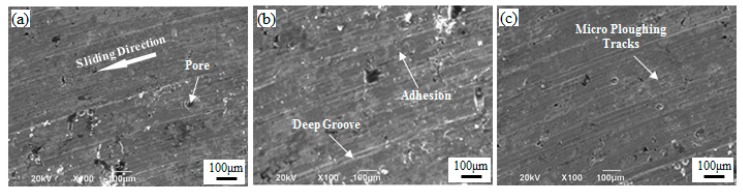
Worn surfaces of the sintered Fe-2Cu-2Mo-0.8C samples added with different forms of Mo: (**a**) Pure Mo powder; (**b**) prealloyed Mo-Fe powder; (**c**) mechanically alloyed Mo-Fe powder.

Figure 10 shows the worn surfaces of the sintered Fe-2Cu-2Mo-0.8C samples added with different forms of Mo. Figure 10a shows the worn surface of the sintered Fe-2Cu-2Mo-0.8C samples added with pure Mo powder, which exhibited some pores and deep grooves parallel to the sliding direction. The SEM image of the sintered samples added with mechanically alloyed Mo-Fe powder exhibited a smooth surface with some micro plowing tracks on the worn surface (Figure 10c). The above features demonstrated that the wear resistance behavior was supported by a higher density and uniform microstructure support. Compared with pure Mo and mechanical alloyed Mo-Fe powder, due to a low density and non-uniform structure (Figure 10b), some deep grooves could be seen in the wear test of the sintered samples added with prealloyed Mo-Fe powder. This was not likely to be retained for a longer time/distance to protect the surface from wear. 

## 4. Conclusions

Fe-2Cu-2Mo-0.8C green compacts with a density of 7.32 g/cm^3^ were obtained by adding with mechanically alloyed Mo-Fe powder to powder mixtures at a pressing temperature of 120 °C and a pressing pressure of 800 MPa. The corresponding sintered specimen had a density of 7.31 g/cm^3^, a hardness of 95 HRB and a tensile strength of 618 MPa, respectively. Compared with adding pure Mo and prealloyed Mo-Fe powder, introducing mechanically alloyed Mo-Fe powder in the powder mixtures was liable to obtain green and sintered specimens with a high density, homogeneous microstructure, finer pearlite and better wear-resistant properties. The present test results have provided a potential way to improve the density and mechanical properties of ferrous materials containing molybdenum.

## Figures and Tables

**Table 1:** Mechanical properties of the sintered Fe-2Cu-2Mo-0.8C specimens added with different forms of Mo.

Mo Addition Form	Tensile Strength (MPa)	Hardness (HRB)
Pure Mo powder	520 ± 11	90 ± 6
Prealloyed Mo-Fe powder	483 ± 7	91 ± 4
Mechanically alloyed Mo-Fe powder	618 ± 5	95 ± 3
